# Enhancing employee wellbeing and happiness management in the wine industry: unveiling the role of green human resource management

**DOI:** 10.1186/s40359-024-01703-y

**Published:** 2024-04-12

**Authors:** Javier Martínez-Falcó, Eduardo Sánchez-García, Bartolomé Marco-Lajara, Luis A. Millán-Tudela

**Affiliations:** Management Department, University of Alicante, Alicante, Spain

**Keywords:** Green human resource management, Sustainable performance, Employee wellbeing, Work engagement, Happiness management, Wine industry, Spain

## Abstract

**Background:**

In today’s business environment, where sustainability has emerged as a strategic axis of business practices, the study of the link between human resources management and environmental management becomes increasingly necessary. In this sense, the present research focuses on analyzing the impact of Green Human Resource Management (GHRM) on the Sustainable Performance (SP) of Spanish wineries, as well as the mediating effect of Employee Wellbeing (EW) and Work Engagement (WE) on this linkage. In addition, age, size and membership in a Protected Designation of Origin (PDO) are introduced as control variables to increase the precision of the cause-effect relationships examined.

**Methods:**

The study proposes a conceptual model based on previous studies, which is tested using structural equations (PLS-SEM) with data collected from 196 Spanish wineries between September 2022 and January 2023.

**Results:**

The findings of the research reveal the existence of a positive and significant relationship between the GHRM development and the SP of Spanish wineries, as well as the partial mediation of EW and WE in this association.

**Conclusions:**

The uniqueness and significance of this study can be attributed to several crucial factors. First, it enhances the understanding and knowledge regarding the advantages associated with GHRM development. Second, no prior research has conducted a comprehensive study on GHRM as a catalyst for SP within the context of Spanish wineries. Third, to the best of the authors’ knowledge, no previous study has analyzed the mediating role of EW and WE as mediators in the relationship between GHRM and SP of wineries.

**Supplementary Information:**

The online version contains supplementary material available at 10.1186/s40359-024-01703-y.

## Background

In contemporary discourse, the imperative of natural resource conservation has risen to the forefront of strategic considerations of senior executives, particularly within the manufacturing industry. This evolving paradigm, elucidated in recent contributions such as Ikram et al. [1] and Younis and Sundarakani [2], underscores a fundamental shift towards the integration of ecological management with financial and social responsibility.

The relevance of this transition is particularly emphasized in the wine industry, which serves as the research context of this study. This sector, symbolic of both tradition and advancement, is at a critical juncture, being especially susceptible to the variations of environmental factors, with global warming and water scarcity emerging as formidable challenges that portend significant implications for its future trajectory [3]. As pointed out by Golicic [4], these environmental hardships are not mere operational obstacles, but existential threats that require a fundamental re-examining of the sector’s practices and strategies.

In this context, Green Human Resource Management (GHRM) represents an exceptionally strategic method for maximizing financial profitability while fulfilling societal and environmental needs where the organization functions [5]. GHRM beyond traditional human resource management boundaries by incorporating a deeply rooted understanding of environmental stewardship into the fabric of a company’s human resource strategies, thereby allowing a transformative impact on how corporations engage with their environmental duties [6]. Indeed, GHRM, by bridging the gap between an organization’s human resource abilities and its sustainability aims and fostering a workforce aware of conservation efforts, guide to enhanced economic, social and environmental performance, in other words enabling improved Sustainable Performance (SP) [7].

At the heart of GHRM lies the principle of aligning business operations with environmental values, an approach that resonates deeply with contemporary employees, who increasingly seek purpose and meaning in their work, as by incorporating green practices into daily routines, from recycling policies to energy efficiency initiatives, organizations not only demonstrate their commitment to sustainability, but also enable employees to feel part of a larger, more meaningful endeavor [8]. This sense of shared purpose and contribution to the greater good fosters greater Employee Wellbeing (EW), as they see their work as an extension of their own values and beliefs [9].

In parallel, GHRM involves the adoption of a more inclusive and participatory approach to people management, where employees are encouraged to take an active part in the formulation and implementation of sustainable practices [10]. This participation not only enriches the work experience, but also strengthens employees’ commitment to the organization, since, by feeling valued and listened to, employees develop a sense of belonging and loyalty to the company, which translates into greater Work Engagement (WE) [11]. In turn, increased EW and WE can lead to greater efficiency, waste reduction and resource optimization of the business, allowing for improvements not only in the environmental footprint of the firm, but also in operational efficiency and long-term corporate viability [12].

In the intricate tapestry of organizational studies, this research strives to illuminate the impact of GHRM practices on the distinctive environment of the wine industry. In particular, the objective of this study specifically centers on examining the impact of GHRM on SP, along with the dual mediation role played by EW and WE within the main GHRM-SP relationship. In this way, the research aims to answer the following three Research Questions (RQs): (1) does GHRM positively influence the SP of wineries? (2) Does EW positively mediate the GHRM-SP relationship? and (3) does WE positively mediate the GHRM-SP relationship? In order to provide answers to these RQs, a theoretical model was examined using structural equation modeling and was tested with primary data gathered from Spanish wineries, collected in the period between September 2022 and January 2023. This analysis addresses a gap in the existing academic literature, as the examination of the relationship between GHRM and SP within the unique context of the Spanish wine industry is an area that has not been previously explored. In fact, the development of the proposed conceptual model provides a robust structure for future research in this field, serving as a basis for generating new scientific knowledge on the subject. Furthermore, the study enriches the understanding of how human resource management practices can be aligned with sustainability and business performance objectives in a key industrial sector, thus offering valuable insights for both academics and wine professionals.

The Spanish wine industry is a relevant context to investigate the impact of GHRM on the SP of wineries, since, beyond its economic importance, with 2.2% of Spain’s total Gross Value Added (GVA), this sector is at the forefront of environmental regulations and evolving ecological and social demands of consumers [13]. Indeed, industry challenges such as global warming and water scarcity require urgent sustainable development strategies, with GHRM being crucial to enhance biodiversity and improve working conditions in winemaking practices for the evolution of this sector towards sustainability [14]. Likewise, by analyzing such research context, the study fills a gap in the existing literature by providing a detailed analysis of GHRM as a catalyst for SP in Spanish wineries and exploring the unknown roles of EW and WE in this dynamic, given that, to the authors’ knowledge, there are no previous studies that have contrasted such a model in the wine industry. Likewise, the wine industry presents itself as a particularly suitable research context for analyzing happiness management due to several unique characteristics of the sector, since, first, the artisanal nature and rich cultural history associated with wine production significantly influence EW and WE [15], and, second, the wine industry, especially in regions with a strong wine identity, offers a collaborative and communal environment, this characteristic being essential for fostering positive working relationships and a sense of belonging, both key elements for employee happiness and well-being [16].

There are several grounds for the necessity and originality of this research. First, it offers new perspectives on the intersectionality of environmental management and human resource practices, especially in the context of SP improvement in Spanish wineries. Second, the results of this research provide valuable information and practical guidance for winery managers seeking to optimize their business performance in its triple dimension. Third, to the best of the authors’ knowledge, there are no previous studies that have exemplified the measuring role of EW and WE jointly in the main GHRM-SP relationship, representing an opportunity to contribute new scientific knowledge. Fourth, the research allows for a unified contribution to both the field of human resource management and corporate sustainability. Fifth, the study enables us to continue expanding the frontiers of knowledge on happiness management in organizations, given that, by identifying the mechanisms through which to improve EW, WE and SP, it favors the generation of a new body of knowledge in an increasingly important field within business management [17–26].

After the introductory remarks, the study moves on to an in-depth examination of theoretical frameworks and hypothesis development. The third part details the methodology, including data acquisition and analysis methods. Part four presents the results of comprehensive structural equation modeling, which uncovers trends and associations in the data. The fifth section examines the results obtained and shows the main conclusions derived from the research; the sixth section discusses the theoretical, practical and policy implications stemming from the research; and finally, the seventh section deals with the limitations, as well as future lines of research to overcome these shortcomings.

## Theoretical underpinning: resource-based view

Within the conceptual framework of GHRM, the Resource-Based View (RBV) presents itself as a crucial theoretical approach for comprehending the dynamics that influence SP in the wine sector. This school of thought emphasizes the significance of internal abilities and organizational skills. These elements are viewed not merely as assets, but as essential facilitators that allow a business to distinguish itself and sustain a competitive advantage, as highlighted by Barney [27]. Particularly in an industry where sustainability has emerged as a key distinguishing factor, understanding how these tangible and intangible resources can be effectively applied to enhance SP is of paramount importance. This understanding, as explored by Martínez-Falcó et al. [28], is vital to the industry’s move toward sustainability, underscoring the role of resources and capabilities in achieving long-term success in the competitive landscape of the wine industry.

Under this approach, GHRM is conceptualized as a distinctive resource, incorporating a series of practices that amplify the ecological and sustainable elements of human resource management, a perspective highlighted by Kim et al. [29]. This approach enhances environmental consciousness and responsibility among employees, subsequently converting this awareness into an organizational capability, an idea elaborated on by Yon et al. [30]. Within the specific environmental setting of wineries, closely tied to land and natural resources, the implementation of GHRM plays a pivotal role in cultivating a competitive edge. This edge is marked by heightened innovation and performance, a concept delineated by Montalvo-Falcón et al. [31]. In addition, the interaction between EW and WE, as a result of environmental practices in environmental management, is fundamental in the GHRM-SP relationship, being able to transform both employee satisfaction and commitment derived from the investment in GHRM into positive sustainable results. Yusliza et al. [32] highlight that when employees are satisfied and engaged, they are more inclined to assume additional responsibilities and surpass expectations. Thus, the RBV approach can provide a comprehensive explanation of how GHRM not only increases winery SP, but also improves EW and WE, which in turn results in improved business performance, highlighting that sustainability in the wine industry is not merely a product of production processes, but is cultivated and reinforced through an informed, engaged and satisfied workforce [33]. The proposed hypotheses, as will be detailed after the description of the variables utilized, further explore this dynamic, underlining the integral role of the GHRM in driving sustainable development in the wine industry.

## Variables conceptualization

The variables employed in this research are: GHRM, WE, EW and SP. First, GHRM refers to the innovative and environmentally conscious merger of human resource management, with the construct focusing on infusing a culture of sustainability into all facets of personnel management [34]. From recruitment to training to performance appraisal, GHRM seeks not only to attract talent aligned with environmental values, but also to foster an active and ongoing commitment to sustainable practices among employees, so by incorporating environmental standards into recruitment processes and job descriptions, GHRM ensures that sustainability is a pillar in organizational identity [35]. Green training programs provide employees with the knowledge and skills necessary to effectively contribute to the company’s environmental goals, reinforcing the evaluation and rewarding of environmental performance the importance of sustainable practices [36] and creating an environment where environmental commitment is not only valued but also essential to professional success [37].

Second, WE is a distinctive psychological state that manifests itself in employees as a combination of dedication and absorption in their work [38]. On the one hand, the dedication translates into deep inspiration and pride in their work, reflecting a meaningful emotional attachment to their work activities [39] and, on the other hand, absorption describes a level of concentration and engagement such that time seems to fly [40], so this deep engagement increases employee satisfaction and well-being, as well as performance and productivity, underlining the importance of fostering a work environment that nurtures these positive psychological aspects [41].

Third, EW relates to the balanced state in the work environment, essential for the optimal functioning of an organization, which is characterized by an environment where effectiveness and team spirit flourish, creating a collaborative and united work space [42]. This well-being is reinforced by a high level of self-esteem and self-confidence among staff, also indicating the presence of low levels of work-related stress and emotional exhaustion [43], which contributes to employees’ resilience and ability to face challenges and creates a positive and motivating work atmosphere [44], crucial for job satisfaction and organizational efficiency [45].

Fourth, SP encapsulates a holistic approach to business success, harmoniously integrating economic prosperity, social commitment and environmental responsibility [46], thus implying a balance between achieving superior financial goals while simultaneously improving the well-being of communities and reducing the ecological footprint [47]. Reflecting a commitment to business ethics and green practices, SP becomes a barometer for companies seeking not only to lead in their industry, but also to forge a responsible path into the future, positioning organizations at the forefront of innovation and social and environmental responsibility [48].

### Green human resource management and sustainable performance

The pivotal role of GHRM in supporting the formulation and sustenance of a business strategy with a focus on sustainability has been emphasized [49]. GHRM is conceived as an essential catalyst in fostering and disseminating an environmentally cooperative culture within organizations [50]. This role is considered crucial, as the adoption of environmental practices within organizations is greatly influenced by aspects including the workforce’s collective efforts, tolerance of mistakes, delivery of environmental training to staff, and thorough assessment and evaluation of environmental objectives [51].

The implementation of GHRM practices is fundamental to instilling an environmentally friendly culture in organizations, with practices ranging from green recruitment and training to performance management and employee engagement playing a crucial role in aligning employee attitudes and behaviors with the organization’s environmental sustainability goals [52]. GHRM thus serves as a bridge between corporate sustainability strategies and employee actions, ensuring that environmental objectives are integrated into the very fabric of human resources policies and practices, this alignment being essential to foster a workforce that is aware of the company’s environmental impact and actively committed to reducing it through innovative and sustainable working practices [53].

Similarly, the influence of GHRM extends beyond internal organizational practices to encompass the broader spectrum of stakeholder engagement and corporate social responsibility, since by integrating environmental values into organizational ethics, companies can enhance their reputation in the eyes of external stakeholders, including customers, investors and regulators, thereby promoting both compliance with regulations and societal expectations, and boosting business competitiveness in an increasingly environmentally conscious marketplace [54]. This strategic focus on sustainability can translate into improved brand image and customer loyalty, as companies are seen as responsible stewards of the environment, making GHRM a key component of building a sustainable brand and securing a competitive advantage in the marketplace [55].

GHRM is acknowledged as a vital contributor to the improvement of a company’s economic performance through the integration of sustainable practices in various organizational areas. The facilitation of these practices has been observed to stem from the green knowledge possessed by employees, which includes reducing energy and water usage, optimizing processes, and managing waste effectively, thereby yielding long-term reductions in operational costs [42]. Consistent with the findings of Carballo-Penela et al. [56], it is recognized that the implementation of eco-friendly solutions can drive innovation in companies, potentially leading to the development of new products, services, and methods of operation, thus opening possibilities for additional revenue. The significance of incorporating sustainable approaches into human resource management, as emphasized by O’Donohue and Torugsa [57], lies in its substantial potential to mitigate risks associated with legal issues, employee dissatisfaction, and environmental challenges, contributing to the protection of company assets and the enhancement of financial performance. Yet, the impact of GHRM extends beyond just economic parameters, as it markedly improves social and environmental performance [58]. El-Kassar and Singh [59] have also noted that aligning GHRM practices with sustainability principles not only aids organizations in achieving short-term financial objectives but also supports their long-term goals of enhancing societal and environmental well-being.

The recruitment of superior talent, facilitated through GHRM, is acknowledged as a fundamental factor in enhancing corporate social performance, leading to an increase in employee satisfaction and the development of better relations with stakeholders [60]. Concurrently, GHRM is instrumental in boosting the environmental performance of companies. This is achieved by aiding the implementation of environmental management practices, which include monitoring natural resources and setting objectives to lessen environmental impact [61]. GHRM also plays a pivotal role in educating employees about improving their environmental conduct at work, often involving the promotion of recycling activities and the adoption of energy-saving measures [62].

Additionally, GHRM assists in the creation of motivational strategies aimed at encouraging environmentally responsible behaviors among employees. These strategies significantly contribute to lowering energy usage, waste reduction, and the diminishment of greenhouse gas emissions, ultimately benefiting both the organization and the environment [63]. The role of GHRM in enhancing a company’s social outcomes through the development of mechanisms that support sustainable and responsible practices has been substantially noted, affecting society broadly and especially targeting employee welfare [64]. The application of GHRM fosters a culture steeped in social responsibility within organizations, a culture that manifests in the conduct and actions of the employees, thereby cultivating a positive and socially responsible corporate identity [65]. The significant influence of GHRM on local communities has been documented by Arnaud and Wasieleski [66], who underscore how socially responsible practices endorsed by companies amplify their connections with the communities they are part of. The influence of GHRM extends even further, impacting the supply chain by ensuring that suppliers adhere to ethical and sustainable practices, thus creating a more positive social impact and preventing harmful practices [67]. Zhang et al. [68] have discussed how GHRM aligns with wider global aims like the United Nations Sustainable Development Goals, signifying a company’s commitment to fulfilling its social responsibilities on an international scale and enhancing its engagement with global societal objectives.

In the field of environmental performance, the crucial role played by GHRM in encouraging the conservation of resources such as energy, water, and materials is recognized. This role significantly contributes to diminishing the environmental footprint and operational expenses of companies [69]. GHRM practices are essential in advocating for approaches that reduce waste generation, including the enhancement of recycling, reuse of materials, and reduction of superfluous packaging, all of which lead to more sustainable operational procedures [70]. Furthermore, GHRM is instrumental in the advancement of sustainable transportation policies, encompassing the implementation of electric vehicles, encouraging carpooling, and promoting the use of bicycles and public transit, all aimed at lowering greenhouse gas emissions among employees [71]. The importance of GHRM in creating measures to evaluate the environmental impact of both operational and human resource practices has been underscored by Zhao and Huang [72]. These measures enable continuous oversight and evaluation of environmental practices, assisting in pinpointing areas needing enhancement, thereby underlining GHRM’s significant contribution to the environmental management within companies [73].

The concept of GHRM has gained considerable attention in recent academic research, particularly regarding its impact on diverse organizational aspects, notably on business performance [74]. Studies in this domain have broadened, exploring GHRM’s influence on various performance metrics across economic, social, and environmental dimensions, often linked with an increased environmental awareness among employees [75]. The research by Rawashdeh [76] is especially noteworthy, establishing a positive correlation between the implementation of GHRM and the long-term success of hospital companies in Jordan. Similarly, Mousa and Othman [77] have demonstrated a cause-and-effect relationship within the healthcare sector in Palestine. Adding to this discourse, recent research by Awwad et al. [78] has provided empirical evidence of the beneficial impact of GHRM in small and medium-sized enterprises in Saudi Arabia, enhancing their economic, social, and environmental performance. This growing body of research underscores the multifaceted benefits of integrating GHRM into organizational strategies.

Despite the academic groundwork laid in analyzing the connection between GHRM and various organizational outcomes, the study of this linkage remains notably limited and warrants further exploration across diverse economic sectors that have yet to be examined. Specifically, to date, there appears to be no existing research that delves into the impact of GHRM on the SP within the winery industry. This gap in knowledge presents a unique and valuable opportunity to deepen our understanding of the subject. The significance of this research avenue is heightened when considering the potential role of GHRM in enhancing the wine industry’s success. GHRM holds the promise not just of enriching biodiversity within this sector but also of improving working conditions and winemaking practices. These improvements are crucial for fostering a sustainable wine industry over the long term. In light of these considerations and with the goal of bridging these identified gaps in scientific understanding, the following hypothesis is proposed:

H1. GHRM has a positive effect on the SP of wineries.

### Green human resource management, employee wellbeing and sustainable performance

GHRM has evolved into a fundamental strategic element, permeating an organization’s operations with an environmental conscience. This integration profoundly influences EW and subsequently the SP of the organization, as explored by Singh et al. [79]. GHRM facilitates the adoption of green hiring practices, serving as a gateway to attract talent that resonates with the organization’s commitment to sustainability and emphasizing that these practices not only emphasize operational efficiency, but the organization’s impact on the natural environment [80].

GHRM is instrumental in shaping an organizational culture that values EW and sustainability because, by integrating environmental values into human resource practices, GHRM fosters a work environment that prioritizes employee health and well-being, with this human resource approach including implementing green practices in the workplace, encouraging work-life balance, and providing opportunities for employees to engage in environmental stewardship [81]. Thus, these practices not only improve employees’ physical and mental well-being, but also instill in them a sense of purpose and fulfillment by aligning their personal values with those of the organization, this alignment being crucial to driving employee engagement, satisfaction and, ultimately, retention [82].

The role of GHRM in fostering EW extends to facilitating an inclusive and participatory work culture, as it actively engages employees in sustainability initiatives and decision-making processes, enabling organizations to create a sense of ownership and empowerment among their employees [83]. This participatory approach enriches the work experience and strengthens employees’ commitment to the organization, as engaged employees are more likely to display pro-environmental behaviors and contribute to the firms’ SP [84].

This alignment of individual and organizational values, according to Hassan et al. [85], is acknowledged in academic discourse as a potent driver of EW, cultivating a sense of belonging and shared purpose among employees [86]. The integration of GHRM practices, therefore, does more than just enhance environmental consciousness within the workplace; it also builds a more cohesive and value-aligned organizational culture, thereby contributing to both the wellbeing of employees and the sustainable advancement of the organization [87].

Green training and participation initiatives are increasingly viewed as crucial mechanisms for empowering employees. They equip staff not only with the skills required for operating in an environmentally conscious work environment, but also offer them opportunities to actively contribute to the company’s sustainability objectives, as noted by Bon et al. [88]. This sense of empowerment and personal involvement in a larger cause is key to fostering deep EW [89]. In this regard, Benevene and Buonomo [90] emphasizes that employee satisfaction stems not only from personal achievements but also from the positive impact their actions have on society and the environment [91]. Furthermore, the alignment of management strategies and compensation with environmental goals is seen as vital for enhancing EW [92]. Such practices underscore the importance of integrating environmental objectives into broader human resource strategies to achieve a more engaged and motivated workforce, committed to both personal and organizational goals [93].

The enhancement of EW through effective GHRM, however, is not merely a goal in itself. In this sense, Alzoubi et al. [94] highlight that satisfied and engaged employees often exceed their basic duties, engaging in innovative problem-solving and demonstrating a heightened commitment to green initiatives, and leading to tangible advancements in a company’s economic, social, and environmental sustainability metrics. The interaction between GHRM, EW and SP is therefore inherently synergistic, as a holistic GHRM approach not only fosters SP through improved operational efficiency and innovation, but also cultivates a work environment in which employees feel valued and integrated into the company’s ecological mission, thereby increasing their satisfaction and, consequently, their contribution to the sustainability of the organization in its three-dimensional framework. Nevertheless, the study of these interrelated variables reveals certain gaps. To date, there has been a lack of research examining EW as a mediating factor in the GHRM-SP relationship. Additionally, the academic literature that simultaneously investigates the catalytic variables of EW and SP is limited, particularly in the sector under examination. To address these gaps, the study puts forward the following research hypotheses:

H2. GHRM has a positive effect on EW of wineries.

H3. EW has a positive effect on SP of wineries.

H4. EW positively mediates the relationship between GHRM and SP of wineries.

### Green human resource management, work engagement and sustainable performance

GHRM emerges as a paradigmatic fusion between environmental sustainability and human capital management, limiting this confluence not only to the improvement of the ecological efficiency of organizations, but also extending to the field of labor commitment, whose influence is crucial in achieving SP in the company [95].

GHRM plays a crucial role in fostering a work environment that is conducive to high levels of employee commitment to environmental sustainability, including incorporating environmental goals into the organization’s mission and values, promoting green behaviors in the workplace, and recognizing and rewarding employee green initiatives [96], thus, by aligning the organization’s environmental goals with employees’ personal values, GHRM cultivates a sense of purpose and belonging, reinforcing employees’ emotional attachment to the organization and their willingness to contribute to its sustainable development goals [97].

Besides, the impact of GHRM on WE extends to enhancing the innovative capabilities of the workforce, as engaged employees, driven by a supportive GHRM framework, are more likely to actively participate in sustainability initiatives, contributing their ideas and creativity, thus driving the organization’s green agenda and, at the same time, fostering a culture of continuous improvement and innovation [98]. Employees thus become agents of change, identifying opportunities for sustainable practices and driving the organization towards green efficiency and effectiveness, benefiting the firms in terms of sustainable performance as well as the personal and professional growth of its employees [99].

At the heart of the relationship between GHRM and WE lies the potential of sustainability practices to engender an ethically sound and healthy work environment. In this respect, Martins et al. [100] argue that the implementation of sustainability policies - such as effective waste reduction and efficient resource management - are a tangible demonstration of environmental responsibility and foster a corporate culture imbued with ecological and ethical awareness. This ethical environment, according to Klingenberg and Kochanowski [101], is a determining factor in increasing WE, thus underpinning a work climate conducive to efficiency and innovation.

In addition, GHRM fosters a sense of purpose and belonging among employees. In this sense, Saeed et al. [102] indicate that employees’ identification with their organization’s sustainable values fuels their loyalty and motivation, not only optimizing internal morale, but also boosting productivity and WE. Tang et al. [103], for their part, highlight that these factors are vital in shaping sustainable organizational results. On the other hand, WE, influenced by GHRM, acts as a catalyst for a SP. In this respect, Saturnino-Neto et al. [104] emphasize that an engaged workforce that is aware of its role in environmental sustainability is a driver of innovation and continuous business performance improvement. Such a contribution manifests itself in an increased organizational capacity to adapt to evolving environmental and social demands while preserving competitiveness and efficiency.

**Fig. 1:**
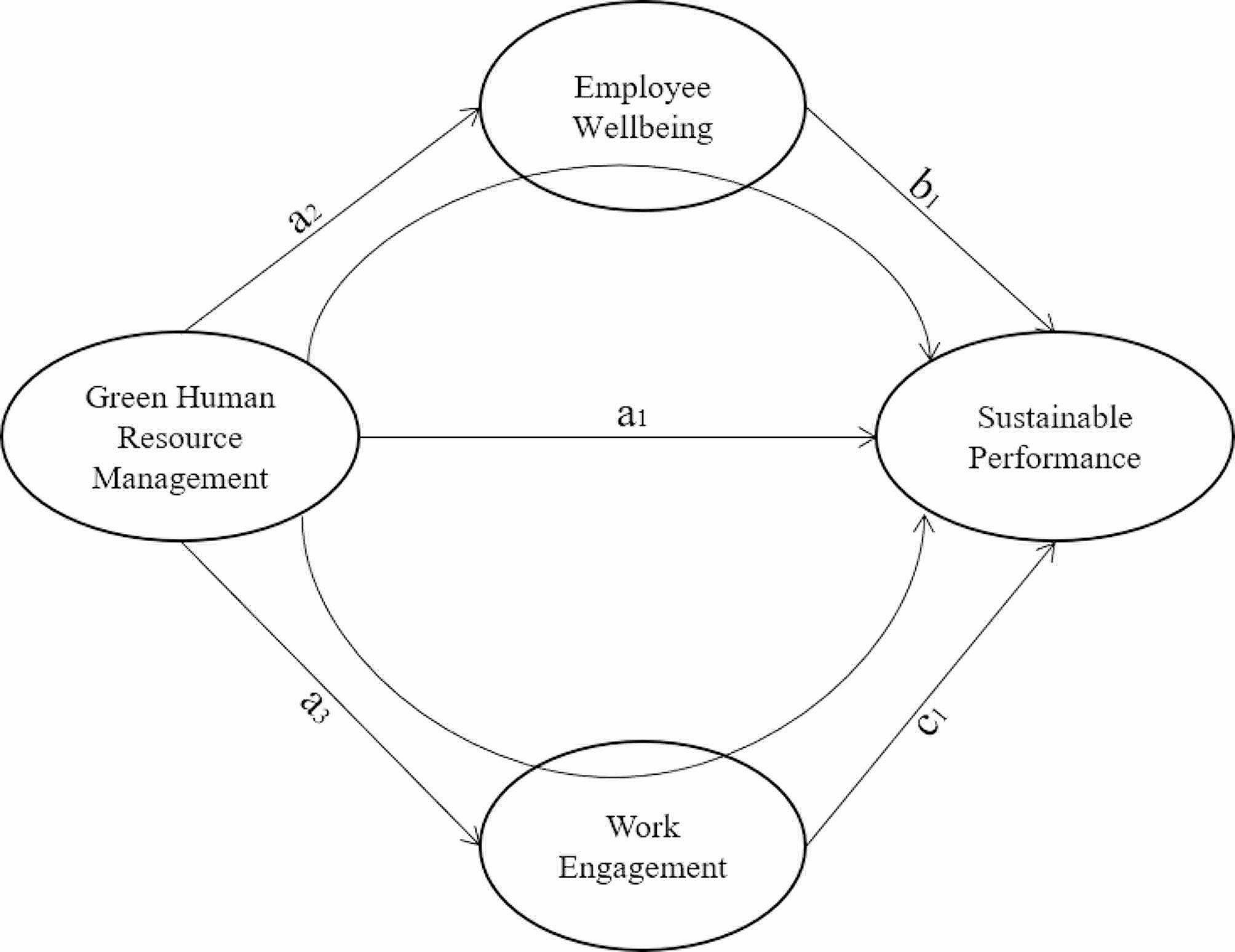
Graphical representation of proposed theoretical model. H1 = a1: Green Human Resource Management ◊ Sustainable Performance. H2 = a2: Green Human Resource Management ◊ Employee Wellbeing. H3 = b1: Employee Wellbeing ◊ Sustainable Performance. H4 = a2 x b1: Green Human Resource Management ◊ Employee Wellbeing ◊ Sustainable Performance. H5 = a3: Green Human Resource Management ◊ Work Engagement. H6 = c1: Work Engagement ◊ Sustainable Performance. H7 = a3 x c1: Green Human Resource Management ◊ Work Engagement ◊ Sustainable Performance. Source: own elaboration

GHRM therefore transcends the mere scope of ethical and responsible personnel management and is a strategic element in the development of sustainable enterprises and the improvement of EW [105]. The synergy created by a sustainable corporate culture and strong labor engagement translates into improved productivity and operational efficiency, both of which are crucial for improving the economic, social and environmental sustainability of any organization in the changing global business landscape [106]. GHRM, therefore, is positioned as an essential component in the architecture of organizations seeking not only to thrive, but also to contribute positively to the environment in which they operate. However, despite the benefits of GHRM as a catalyst for agricultural improvement, there are some gaps around its study. First, to the authors’ knowledge, there are no previous studies that have analyzed the mediating role of EW in the GHRM-SP relationship. Second, there is a limited amount of research that has examined the catalytic variables of EW in the sector under study. In order to overcome these shortcomings, the following hypotheses are proposed (see Fig. 1):

H5. GHRM has a positive effect on WE of wineries.

H6. WE has a positive effect on SP of wineries.

H7. WE positively mediates the relationship between GHRM and SP of wineries.

## Methods

The research is systematically structured into six principal segments: (1) the context of the research is outlined, (2) the sample and population are defined, (3) the constructs under consideration are identified, (4) the control variables employed in the study are specified, (5) the methodology for analysis is delineated and (6) study bias is controlled. This organization aims to facilitate a comprehensive comprehension of the research process. In-depth elucidations of each segment are furnished in the ensuing descriptions.

### Research context

This research is contextualized in the Spanish wine sector, choosing this context of analysis for a series of reasons that justify its suitability for this analysis. Initially, the sector’s significant economic impact in 2022 cannot be overlooked, contributing over €23.7 billion to Spain’s GVA, which represents 2.2% of the nation’s total GVA [107]. Furthermore, the relevance of GHRM insights for the wine industry in addressing contemporary ecological and social expectations of consumers, in conjunction with the stringent environmental regulations it confronts, underscores the critical and timely nature of this investigation within the industry’s context [31]. Additionally, the Spanish wine sector is acknowledged for its substantial environmental and heritage value [108], which heightens the importance of examining the drivers of SP to enhance the industry’s environmental sustainability [109]. Lastly, this research pioneers by exploring the intricate relationship between human resource management and sustainability in the wine business domain, introducing a novel theoretical framework to analyze these interconnections.

## Population and sample collection

This study is characterized by its focus on wineries classified in segment 1102 of the National Code of Economic Activities (CNAE, for its acronym in Spanish). Using the Iberian Balance Sheet Analysis System (SABI, for its acronym in Spanish) database, a list of 4,373 wineries was compiled, this being the population under study. A questionnaire, developed after an extensive review of the academic literature, was used for data collection. Prior to use, the questionnaire was pre-tested, involving experts in environmental management, quality control and experienced winemakers, to determine its accuracy and relevance. This exhaustive pre-test was crucial to ensure the instrument’s effectiveness in accurately capturing the intended constructs, thus improving the reliability and validity of subsequent data. Data collection, conducted via a questionnaire managed through Qualtrics, ran from September 2022 to January 2023. To maintain data integrity, responses were solicited from CEOs, recognized for their strategic vision and extensive knowledge of operations, and this method was chosen in order to obtain detailed and informed information pertaining to the strategic apex of the wineries, resulting in 196 valid responses from 196 different winery executives. In addition, it should be noted that informed consent to participate was obtained from all study participants. The sample included representatives from all Spanish autonomous communities, with significant contributions from the regions of Castilla y León (14.36%), Catalonia (13.37%), Castilla La-Mancha (10.89%) and La Rioja (9.90%), ensuring a geographically broad and representative sample.

### Variable measurement

In the conducted study, to maintain consistency, reliability, and validity, scales that have undergone prior validation were utilized for variable measurement (see Appendix). The GHRM variable was evaluated through a scale formulated by Mousa and Othman [77]. This scale includes three first-order variables: green hiring, which is comprised of 6 items; green training and involvement, consisting of 8 items; and green performance management and compensation, also comprising 8 items. The EW construct was evaluated using a scale with 7 items, formulated by Alimo-Metcalfe et al. [110]. The WE variable, on the other hand, was measured based on a 9-item scale utilized by Gim et al. [111]. Moreover, the SP construct was gauged using an integrated scale derived from the works of Wang and Wang [112], Paulraj [113], and Paillé et al. [114], combining dimensions such as economic performance (4 items), social performance (6 items), and environmental performance (5 items), thus providing a comprehensive evaluation of various aspects of business performance. All scales employed 7-point Likert-type measurements, conceptualizing the main variables as reflexive constructs. Likewise, the 95% confidence intervals for each of the correlations were tested using a bootstrap test with 5,000 subsamples.

### Control variables

Control variables in the study, including the age and size of the winery, as well as affiliation with a PDO, were also integrated. The PDO variable was treated as dichotomous, being assigned a value if the winery was affiliated with one or more PDOs, and no value otherwise. The size of the winery was quantified in accordance with the standards established by the Organization for Economic Co-operation and Development [115], and the age of the wineries was determined based on the period from their establishment until 2023. This methodological rigor and comprehensive approach formed the foundation for the constructs and measures used in this academic investigation. Furthermore, it should be noted that the questionnaire was prepared expressly for this study and can be reviewed in the Appendix.

### Analytical approach

In the conducted study, the analysis was characterized by employing partial least squares structural equation modeling (PLS-SEM), using SmartPLS v. 4.0.0 software (see Table 1). PLS-SEM is acknowledged as an effective statistical technique that enables the exploration of complex theoretical relationships among variables, including those that are latent. This method is particularly relevant in management research, where constructs frequently possess an abstract nature [116]. The choice to use PLS-SEM was influenced by several crucial considerations. Initially, the complexity of the GHRM construct required a method capable of adeptly managing multivariate scenarios, a known forte of PLS-SEM [117]. In this sense, it should be noted that, although the PLS-SEM methodology can be adapted for confirmatory purposes [116], the main orientation of this study focuses on the exploration and modeling of complex relationships between theoretical constructs, since the study aims to examine direct interactions and mediators in a research context, the wine industry, where the underlying theories are still under development, which implies a need for methodological flexibility that goes beyond the typical limits of a confirmatory approach. Thus, the analysis takes advantage of the potential of PLS-SEM for theoretical exploration and modeling of complex relationships, in a research context where discovery and the generation of new knowledge take precedence over the confirmation of pre-existing theories. Additionally, the existence of explicit interrelations among variables necessitated an analytical approach that could address both direct and mediated relationships within a comprehensive model simultaneously, a characteristic associated with PLS-SEM [118]. Furthermore, the robustness of the sample size, surpassing the recommended minimum of 100 observations as indicated by Reinartz et al. [119], confirmed the suitability of PLS-SEM for this research. The strategic application of PLS-SEM enabled the study to delve into intricate theoretical constructs and relationships among variables, thereby enhancing the precision and depth of the findings in the field of management research.

**Table 1:** Methodology data sheet

Category	Study values/information
CNAE Code	1102
Population	4,373
Sample	196
Questionnaire management software	Qualtrics
Sample collection period	September 2022 - January 2023
Constructs employed	GHRM, EW, WE and SP
Control variables	Age, size and PDO
Contrast method	PLS-SEM
Contrast method software	SmartPLS v. 4.0.0

Source: compiled by the authors

### Bias control

In addressing bias in this study, a comprehensive approach was taken to mitigate potential risks, employing both procedural and statistical measures. Procedurally, emphasis was placed on anonymity and the nonjudgmental nature of the survey to reduce social desirability bias and participant discomfort. From a statistical point of view, first, the application of Harman’s single factor test revealed a dominant factor accounting for only 22.5% of the total variance, significantly below the 50% benchmark, indicating a negligible presence of common method bias; second, to address potential nonresponse bias, a comparative examination was conducted between the original sample of 211 respondents and the final cohort of 196 through a t-test, with this comparison showing no significant variations in demographics or responses, as the p-values exceeded the 0.05 mark at the 95% confidence level, suggesting that nonresponse bias was not a concern; and; third, the research included tests for homogeneity of variances, absence of multicollinearity and construct validity, with Levene’s test confirming homogeneity of variances (F = 1.24, *p* = 0.27), with multicollinearity considered unproblematic as correlation coefficients below 0 were obtained, 7 and Variance Inflation Factor (VIF) scores below 5 and further validating the confirmatory factor analysis constructs by showing factor loadings above 0.5 and acceptable fit indices, which together underline the methodological integrity of the study and the credibility of its conclusions.

## Results

Following the methodology suggested by Hair et al. [120], the results of this research were systematically divided into two main segments: firstly, the assessment of the measurement model, and secondly, the examination of the structural model. The initial phase encompassed an evaluation of the model’s overall fit. As illustrated in Table 2, the model’s overall fit is considered satisfactory, as indicated by the Standardized Root Mean Square Residual (SRMSR) being lower than the established threshold of 0.080. This indicates the model’s adequacy in accurately representing the relationships it is designed to measure, in line with the standards set forth by Hu and Bentler [121]. Furthermore, the unweighted least squares discrepancy (d_ULS) and the geodesic discrepancy (d_G) were examined post-bootstrapping to ensure they remained within the prescribed confidence intervals, specifically beneath the HI95 and HI99 benchmarks.

**Table 2:** Overall model fit

	Value	HI95	HI99
SRMR	0.036	0.057	0.074
d_ULS	0.341	0.545	0.728
d_G	0.594	0.717	0.887

Source: compiled by the authors.

In the assessment of the measurement model, Table 3 presents the individual reliability of each item constituting the constructs under investigation. These items demonstrate reliability as their loadings surpass the minimum threshold of 0.707, a benchmark established by Carmines and Zeller [122]. The table further elucidates findings pertaining to internal consistency and convergent validity. For internal consistency, the criteria are fulfilled with Cronbach’s alpha, composite reliability (Pc), and the Dijkstra-Henseler criterion (Pa) values all exceeding 0.8, as suggested by Hair et al. [120]. This indicates a strong association among indicators within the same construct. Regarding convergent validity, as indicated in the table, the constructs under examination meet this criterion. This is evidenced by the Average Variance Extracted (AVE) for each variable surpassing the 0.5 level, signifying that each construct accounts for more than half of the variance in its indicators.

**Table 3:** Assessment of item reliability, convergent validity and discriminant validity

Construct/Items	Outer Loadings	Rho_c (Pc)	Rho_a (Pa)	Cronbach’s Alpha		AVE
Green Human Resource Management (GHRM)		0.882	0.845		0.802	0.625
GHRM 1	0.745					
GHRM 2	0.864					
GHRM 3	0.801					
Employee Wellbeing (EW)		0.905	0.885		0.814	0.687
EW 1	0.801					
EW 2	0.788					
EW 3	0.827					
EW 4	0.864					
EW 5	0.841					
EW 6	0.786					
EW 7	0.741					
Work Engagement (WE)		0.901	0.895		0.841	0.625
WE 1	0.849					
WE 2	0.846					
WE 3	0.887					
WE 4	0.834					
WE 5	0.815					
WE 6	0.778					
WE 7	0.789					
WE 8	0.718					
WE 9	0.895					
WE 10	0.741					
Sustainable Performance (SP)		0.896	0.848		0.815	0.614
SP 1	0.789					
SP 2	0.756					
SP 3	0.898					

Note: The indicators for the second-order variables are: GHRM 1 = Green

Hiring; GHRM 2 = Green Training and Involvement; GHRM 3 = Green Performance Management and Compensation; SP 1 = Economic Performance; SP 2 = Social Performance; SP 3 = Environmental Performance.

Source: compiled by authors.

For the assessment of discriminant validity, the Heterotrait-Monotrait (HTMT) criterion was utilized, with the results presented in Table 4 showing values significantly surpassing 0.85. This indicates that each construct is distinctly separate from the others, effectively capturing and measuring diverse phenomena. Moreover, the 95% confidence intervals for each correlation were examined using bootstrapping. This verification process affirmed that the criteria for discriminant validity were maintained, demonstrating a distinct differentiation among the constructs.

**Table 4:** Discriminant validity analysis based on the HTMT criterion

	AGE	SIZE	PDO	GHRM	SP	EW	WE
AGE							
SIZE	0.045						
PDO	0.136	0.214					
GHRM	0.046	0.371	0.146				
SP	0.079	0.048	0.045	0.347			
EW	0.124	0.114	0.147	0.147	0.469		
WE	0.048	0.047	0.249	0.241	0.354	0.707	

Source: compiled by authors

**Fig. 2:**
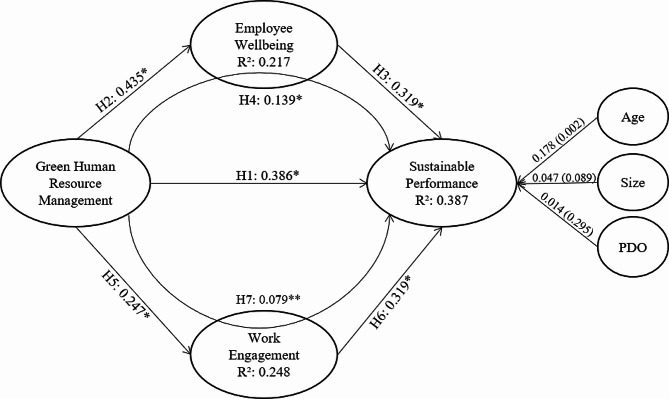
Theoretical model with R-squared, path coefficients (β) and significance. Notes: *p < 0.001 **p < 0.05. Source: own elaboration.

Subsequent to the assessment of the measurement model, the structural model was examined, as illustrated in Fig. 2. This figure displays data related to path coefficients and the R-Squared values obtained from a bootstrap analysis involving 5,000 subsamples. The findings indicate a significant positive influence of GHRM on SP, including the proposed mediating relationships, since it is observed that GHRM impacts SP both directly and through indirect pathways via EW and WE. The direct effect is quantified at 0.386, while the indirect effects are recorded at 0.139 and 0.079, all exhibiting statistical significance. The total effect of GHRM on SP stands at 0.604.

These results enable the confirmation of all seven proposed hypotheses. They demonstrate a significant positive effect of GHRM on SP (H1, β = 0.386; *p* < 0.000), of GHRM on EW (H2, β = 0.435; *p* < 0.000), of EW on SP (H4, β = 0.319; *p* < 0.000), of GHRM on WE (H5, β = 0.247; *p* < 0.000), and of WE on SP (H6, β = 0.319; *p* < 0.000). Additionally, EW is found to partially mediate the GHRM-SP relationship (H4, β = 0.139; *p* < 0.000), and WE partially mediates the GHRM-SP link (H7, β = 0.079; *p* < 0.025). The data highlight GHRM’s strongest effect being on EW, with WE emerging as a crucial predictor of SP. Regarding control variables, the findings suggest that while organizational size has a positive and significant effect on SP (β = 0.178; *p* < 0.002), age (β = 0.047; *p* < 0.089) and PDO membership (β = 0.014; *p* < 0.295) exert a positive, albeit non-significant, impact on the dependent variable, as further detailed in Table 5.

**Table 5:** Evaluation of the structural model

Direct Effects	Path Coefficient	t-Value	P Values	95% BCCI	Hypothesis supported
GHRM ◊ SP	0.386	4.505	0.000*	[0.245; 0.486]	H1 supported
GHRM ◊ EW	0.435	5.014	0.000*	[0.361; 0.567]	H2 supported
EW ◊ SP	0.319	3.768	0.000*	[0.217; 0.447]	H3 supported
GHRM ◊ WE	0.247	2.984	0.000*	[0.138; 0.475]	H5 supported
WE ◊ SP	0.319	3.879	0.000*	[0.205; 0.426]	H6 supported
Indirect Effects	**Path Coefficient**	**t-Value**	**P Values**	**95% BCCI**	**Hypothesis supported**
GHRM ◊ EW ◊ SP	0.139	2.859	0.000*	[0.078; 0.236]	H4 supported
GHRM ◊ WE ◊ SP	0.079	2.108	0.025**	[0.025; 0.189]	H7 supported

Notes: BCCI = Bias Corrected Confidence Intervals; *p < 0.001 **p < 0.05

Source: compiled by authors

In the concluding phase of the analysis, the model’s quality was evaluated utilizing the Geisser test (Q2). Following the criteria established by Hair et al. [118], it was determined that for a model to be considered acceptable and to have predictive relevance, the Q2 values must surpass 0. Thresholds exceeding 0.250 and 0.500 signify medium and large predictive relevance, respectively. As demonstrated in Table 6, the model is shown to possess medium predictive relevance, as evidenced by Q2 values exceeding the 0.250 benchmark.

**Table 6:** Construct cross validated redundancy

	SSO	SSE	Q² (= 1-SSE/SSO)
AGE	196	196	
EW	1176	957.245	0.295
GHRM	854	588	
WE	1568	1142.182	0.298
PDO	196	196	
SIZE	196	196	
SP	854	347.745	0.305

Source: compiled by authors.

## Conclusions

The present research provides empirical evidence of the positive relationship between the development of GHRM and the SP achieved by Spanish wineries, shedding light on the transformative impact of this typology of human resource management in stimulating EW and EW and how these, in turn, can lead to improved SP.

The findings of the study therefore allow to answer the three RQs posed. Regarding the first RQ, the research unravels the positive relationship between GHRM and SP, demonstrating that the adoption of green practices in human resource management significantly improves the SP of these wineries, which underlines the strategic importance of incorporating environmental considerations into huma resource policies, not only for environmental improvement but also for tangible enhancements in organizational performance. As for the second RQ, the research elucidates the mediating role of EW in the GHRM-SP dynamic, thus revealing that the implementation of GHRM positively influences EW, which in turn contributes to SP. This mediation effect is particularly insightful, as it underscores the critical importance of EW in achieving sustainable outcomes, suggesting that the path to sustainable performance goes through both direct environmental initiatives and improved EW. Concerning the third RQ, it is empirically demonstrated that GHRM positively influences WE, which in turn leads to improved sustainable performance, thus underlining the importance of engaging employees through GHRM practices as a key strategy to achieve sustainability goals.

The results of this study present a significant alignment with recent research conducted by Martínez-Falcó et al. [104] and Montalvo-Falcón et al. [26], who have highlighted the fundamental role of green knowledge stocks and GHRM in improving the business performance of Spanish wineries, both studies underscoring the growing recognition of environmental management as a strategic asset in the business environment, especially in sectors intimately related to natural resources and sustainability, such as the wine sector. Both Martínez-Falcó et al. [104] and Montalvo-Falcón et al. [26] have effectively demonstrated that the integration of ecological knowledge into business practices, together with the implementation of GHRM policies, contributes significantly to the overall performance of wineries, with this synergy of ecological knowledge and GHRM creating a framework in which environmental considerations are seamlessly woven into the fabric of the organization’s culture and operations.

Nonetheless, a critical observation that emerges from the present research is the lack of exploration in previous studies on the mediating effects of EW and WE on the dynamic relationship between GHRM and entrepreneurial performance, given that, while previous research has established the direct impact of GHRM on entrepreneurial performance, this study extends the discourse by introducing EW and WE as crucial mediating variables, which introduces a nuanced understanding of how GHRM, beyond its direct influence, can indirectly improve business performance through its positive effects on EW and WE. This extension of the existing literature is particularly noteworthy as it underscores the human element in the relationship between sustainable practices and business performance, since, by focusing on EW and WE, the study clarifies how employee satisfaction, morale and commitment are influenced by GHRM practices, which in turn shape broader business outcomes. This research therefore not only enriches the theoretical understanding of the link between sustainability and business performance, but also offers practical recommendations for organizations seeking to leverage the full spectrum of benefits associated with GHRM as addressed in the following section.

### Theoretical, practical and policy implications

From the results of this research, several implications have emerged, encompassing theoretical, practical, and policy aspects. Theoretically, the study enhances the existing academic literature on human resource management by providing empirical insights specific to the Spanish wine industry. It establishes the notable and positive influence of GHRM on SP, while elucidating the mediating roles of EW and WE in this dynamic. This study, therefore, extends the understanding of EW and WE, as well as their significance in enhancing business performance, which can be theoretically underpinned by the RBV theory. Likewise, the research contributes to the literature on happiness management in the business context by focusing on the intersection of GHRM and EW in the wine industry, providing a new perspective by analyzing how environmentally sustainable practices in human resource management can positively influence employee happiness and engagement, since, by integrating GHRM into business operations, the study demonstrates operational efficiency, cost savings, improved EW and increased WE organization.

In terms of practical implications, first, the study highlights the economic benefits of integrating GHRM into business operations. For wine managers, this translates into potential cost savings and increased operational efficiency through reduced energy and water consumption and process optimization. In addition, the adoption of green solutions can lead to the development of innovative products and services, creating new sources of revenue. Therefore, winery managers can leverage these benefits to improve their competitive advantage and market positioning. Second, the social and environmental impact of GHRM is significant, so managers are encouraged to consider how GHRM can strengthen their corporate social performance and improve stakeholder relations, which involves not only hiring talent aligned with sustainability goals, but also implementing environmental management practices that can reduce the winery’s environmental footprint. Such practices align the winery with growing consumer and regulatory expectations for environmental responsibility. Third, the study highlights the importance of EW and WE as mediators in the relationship between GHRM and sustainable performance. For winery managers, this means fostering a workplace culture that values employee involvement in sustainability initiatives, since by empowering employees through green training and engagement, managers can improve EW, leading to greater engagement and productivity. Fourth, from a strategic standpoint, the study places GHRM as a key component of sustainable business development. For wine managers, this implies that GHRM should be viewed not just as a set of practices, but as a comprehensive strategic approach to achieving sustainable performance, involving aligning human resource strategies with environmental sustainability goals to foster a workforce that is both environmentally conscious and innovative. Fifth, the study underscores the crucial value of GHRM for winery managers to properly manage happiness in organizations, given that by integrating GHRM practices, wineries not only achieve operational efficiency, but also promote a work environment where employees feel valued and part of a larger effort towards sustainability, which can translate into an alignment of individual and organizational values with sustainability goals, thus creating a more satisfying and motivating work culture.

In terms of policy implications, the study emphasizes the critical juncture facing the wine industry due to environmental challenges such as global warming and water scarcity, which calls for a fundamental reassessment of industry practices and strategies. This highlights the need for policy frameworks that support and encourage sustainable practices in industries vulnerable to environmental change. Likewise, the potential role of GHRM in biodiversity enrichment, improved working conditions and winemaking practices points to a broader policy discourse on sustainable development in business operations, suggesting the need for policy support to encourage sustainable practices in various economic sectors. Similarly, incorporating GHRM practices, such as reducing energy and water consumption and waste management, aligns with broader policy goals of environmental conservation and sustainable resource management. This alignment implies political responsibility to facilitate and promote such practices through supportive policies and incentives. Furthermore, the study’s focus on improving EW and WE through GHRM underscores the importance of policy initiatives that encourage companies to adopt practices that improve employee satisfaction, commitment and engagement, thereby contributing to the overall well-being of society.

### Limitations and future lines of research

While the study presents insightful findings, it is essential to recognize its limitations. Expanding the scope of the research to include various globally renowned wine regions could enhance its significance, allowing for a comparative analysis of human resource practices in both Old and New World wineries. Further exploration into factors such as winery size, historical context, and PDO affiliation would also be beneficial, as these elements were only incorporated as control variables in the current study. Conducting a multi-group analysis could be instrumental in uncovering potential variations within the proposed model based on these factors. Moreover, this research primarily focuses on the roles of EW and WE as mediators in the relationship between GHRM and SP, which underscores the need for future research to examine additional mediating factors that could be critical to understanding this relationship more comprehensively.

### Electronic supplementary material

Below is the link to the electronic supplementary material.

Supplementary Material 1

## Data Availability

No datasets were generated or analysed during the current study.
