# Correction: Enhancing employee wellbeing and happiness management in the wine industry: unveiling the role of green human resource management

**DOI:** 10.1186/s40359-024-01776-9

**Published:** 2024-05-21

**Authors:** Javier Martínez-Falcó, Eduardo Sánchez-García, Bartolomé Marco-Lajara, Luis A. Millán-Tudela

**Affiliations:** Management Department, University of Alicante, Alicante, Spain

**BMC Psychology (2024) 12:203**

10.1186/s40359-024-01703-y

Following publication of the original article, it came to the journal’s attention that ‘Full professor’ had been erroneously included in the author list as an author. This formatting error has since been corrected in the article. The publisher thanks you for reading and apologizes for any inconvenience caused.

