# Supplementary material for: Enhancing employee wellbeing and happiness management in the wine industry: unveiling the role of green human resource management
Source: BMC Psychol. 2024 Apr 12;12:203. doi: 10.1186/s40359-024-01703-y (PMC11015661; doi:10.1186/s40359-024-01703-y)
Supplement: Supplementary file 1 — Supplementary Material 1 [file 40359_2024_1703_MOESM1_ESM.docx]

# **Appendix**

*A1.* Composition of the study variables

| **Variable** | | **Questions/items** | **Authors** |
| --- | --- | --- | --- |
| Control Variables (CV) | | CV 1. Is the winery adhered to at least one Protected Designation of Origin? | Martínez-Falcó et al. [71] |
|  |  | CV 2. When was the winery founded? | Martínez-Falcó et al. [71] |
|  |  | CV 3. How many employees does the winery have? | OECD [79] |
| Green Human Resource Management (GHRM) | Green Hiring (GH) | GH 1. The organization prefers to recruit employees that have knowledge about environment | Mousa and Othman [49] |
|  |  | GH 2. Applicants for jobs in the organization are subject to interviews to test their knowledge about environment |  |
|  |  | GH 3. In addition to other criteria, employees are selected based on environmental standards |  |
|  |  | GH 4. Job seekers are attracted by the environmental image and policies of the organization |  |
|  |  | GH 5. The job description includes the job’s environmental aspects |  |
|  |  | GH 6. The recruitment message includes organizations’ environmental values in job advertisement |  |
|  | Green Training and Involvement (GTI) | GTI 1. Training programs about environment are provided to large-scale individuals in the organization |  |
|  |  | GTI 2. In general, staff are satisfied with the organization’s green training |  |
|  |  | GTI 3. Topics offered through green training are modern and suitable for the institution’s activities |  |
|  |  | GTI 4. The organization provides formal environmental training programs for employees to increase their ability to promote them |  |
|  |  | GTI 5. Environmental training is a priority and an important investment |  |
|  |  | GTI 6. The need assessment for green training helps to familiarize employees with environmental practices |  |
|  |  | GTI 7. Evaluation of green training and development helps to measure the employees’ level of green knowledge and awareness |  |
|  |  | GTI 8. Environmental objectives contain green training and development aspects |  |
|  | Green Performance Management and Compensation (GPMC) | GPMC 1. Specific environmental goals are adopted by every manager and employee in the organization |  |
|  |  | GPMC 2. When environmental programs are improved, employees are rewarded for their remarkable ideas |  |
|  |  | GPMC 3. Employees who have achieved or exceeded the objectives of the environmental institution are rewarded with non-cash equivalents or other cash prizes |  |
|  |  | GPMC 4. Section managers reward staff in their departments when they improve environmental programs |  |
|  |  | GPMC 5. Environmental performance is recognized in public |  |
|  |  | GPMC 6. One of the criteria employee performance assessment is the achievement of environmental objectives |  |
|  |  | GPMC 7. There are adequate assessments of staff performance after attending courses on environmental topics |  |
|  |  | GPMC 8. Employees are punished for non-compliance with environmental standards in the organization |  |
| Employee Wellbeing (EW) | | EW 1. There is a high sense of fulfilment among staff | Alimo-Metcalfe et al. [74] |
|  |  | EW 2. There is a high level of self-esteem among staff |  |
|  |  | EW 3. There is a high level of self-confidence |  |
|  |  | EW 4. There is a low level of job-related stress |  |
|  |  | EW 5. There is a low level of job-related emotional exhaustion |  |
|  |  | EW 6. There is a strong sense of team spirit |  |
|  |  | EW 7. There is a strong sense of team effectiveness |  |
| Work Engagement (WE) | | WE 1. Employee involvement and participation are designed to maximize employees’ performance | Gim et al. [75] |
|  |  | WE 2. At my work, I feel bursting with energy |  |
|  |  | WE 3. At my job, I feel strong and vigorous |  |
|  |  | WE 4. I am enthusiastic about my job |  |
|  |  | WE 5. My job inspires me |  |
|  |  | WE 6. When I get up in the morning, I feel like going to work |  |
|  |  | WE 7. I feel happy when I am working intensely |  |
|  |  | WE 8. I am proud of the work that I do |  |
|  |  | WE 9. I am immersed in my work |  |
|  |  | WE 10. I get carried away when I am working |  |
| Sustainable Performance (SP) | Economic Performance (EP) | EP 1. Our company's average return on investment is above the industry average over the past five years | Wang and Wang [76] |
|  |  | EP 2. Our company's average profit is above the industry average over the last five years |  |
|  |  | EP 3. Our company's profit growth is above the industry average over the last five years |  |
|  |  | EP 4. Our company's average sales profitability is above the industry average over the last five years |  |
|  | Social Performance (SOP) | SOP 1. Our company has improved the well-being of its stakeholders compared to its competitors over the last five years | Paulraj [77] |
|  |  | SOP 2. Our company has improved the health and safety of the community in which it operates relative to its competitors over the past five years |  |
|  |  | SOP 3. Our company has reduced its environmental impact and risks to the general public relative to its competitors over the past five years |  |
|  |  | SOP 4. Our company has improved the occupational health and safety of employees relative to its competitors over the past five years |  |
|  |  | SOP 5. Our company has protected the claims and rights of its stakeholders relative to its competitors over the past five years |  |
|  | Green Performance (GP) | GP 1. Our company has reduced waste and emissions from operations relative to its competitors over the past five years | Paillé et al. [78] |
|  |  | GP 2. Our company has reduced the environmental impact of its products/services relative to its competitors over the past five years |  |
|  |  | GP 3. Our company has reduced its environmental impact by establishing partnerships with its competitors over the last five years |  |
|  |  | GP 4. Our company has reduced the risk of environmental accidents, spills and emissions compared to its competitors in the last five years |  |
|  |  | GP 5. Our company has reduced purchases of non-renewable materials, chemicals and components relative to its competitors over the past five years |  |

Source: own elaboration
